# The Influence of Episodic Future Thinking on Prospective Memory in Older Adults

**DOI:** 10.3390/bs14121171

**Published:** 2024-12-06

**Authors:** Zhanyu Ma, Xinyuan Zhang

**Affiliations:** 1 School of Psychology, Northeast Normal University, Changchun 130024, China; 18764720275@163.com; 2 Jilin Provincial Key Laboratory of Cognitive Neuroscience and Brain Development, Changchun 130024, China

**Keywords:** prospective memory, episodic future thinking, older adults, delay interval

## Abstract

Previous research has demonstrated that episodic future thinking (EFT) can enhance prospective memory (PM); however, its effects on older adults have been less explored. This study examines the impact of EFT training on PM in both older and younger adults under varying delay intervals. Experiment 1 employed a 2 (EFT training: present vs. absent) × 2 (age: younger adults vs. older adults) × 2 (delay interval: 5 min vs. 20 min) between-subjects design. The results revealed a significant main effect of EFT training (*p* < 0.001), indicating that such training improves PM performance. Among younger adults, a significant difference in PM performance was found between the trained and untrained groups (*p* = 0.03), while among older adults, this difference was only marginally significant. This suggests that the facilitative effect of EFT training is more pronounced in younger adults. Additionally, there was a significant main effect of delay interval (*p* = 0.01), with shorter intervals yielding better PM performance than longer intervals. Experiment 2 focused on the impact of specificity in EFT training on PM in both age groups. A 2 (training: specific vs. non-specific) × 2 (age: younger vs. older adults) × 2 (delay interval: 5 min vs. 20 min) between-subjects design was used. Results indicated that older adults in the specific training group outperformed those in the non-specific training group (*p* = 0.03), whereas no difference was observed among younger adults. This finding suggests that specific training is more effective for enhancing prospective memory in older adults. Moreover, older adults exhibited differences based on the delay interval, with a 20 min interval impairing performance (*p* = 0.04), while younger adults showed no difference between the two intervals. These findings will be discussed in relation to the Multiprocess Model and the Preparatory Attention and Memory Processes Theory.

## 1. Introduction

Prospective memory (PM) is the ability to remember to execute a planned action at a specific time or in response to external cues [1,2]. This function is one of the most frequently relied upon in daily life, as it allows us to carry out intentions without constant reminders. Based on the type of cues, prospective memory is typically categorized into event-based prospective memory (EBPM) and time-based prospective memory (TBPM) [3,4]. On one hand, EBPM involves remembering to perform an intended action when a specific cue appears or a designated event occurs, such as remembering to buy a loaf of bread when passing a bakery. On the other hand, TBPM requires remembering to carry out an intended action at a specific time or after a set duration, such as remembering to attend a meeting at 3 p.m. or to pick up the children from school after three hours. Research on adults has shown that PM performance declines with age [5]. This decline often leads to frequent PM errors among older adults, sometimes with serious, even life-threatening consequences [6]. For example, an older adult who needs to take hypoglycemic medication 30 min before meals could experience health complications if they forget to take it on time. Therefore, it is particularly important to explore effective strategies to enhance PM in older adults.

In recent years, there has been growing research interest in using episodic future thinking (EFT) as an encoding strategy to enhance PM performance [7,8,9,10]. Similar to PM, EFT is a forward-looking cognitive ability, as it involves imagining future events and one’s role within these scenarios based on current or past experiences [11,12]. Reflecting this connection, studies have found a significant relationship between EFT and PM [13,14,15,16,17]. For instance, EFT training has been shown to enhance PM performance across different age groups, including adults [18,19], adolescents [9], and children [8,10]. In one example, Neroni et al.’s experiment [18], adults who received EFT training were asked to mentally simulate PM tasks that needed to be completed the following day. The results indicated that participants who received EFT training outperformed those who did not. Moreover, extensive evidence from neuroimaging studies suggests that EFT and memory rely on similar psychological and neural processes. What is more, greater similarity in brain activation patterns between encoding (imagining future scenarios) and retrieval stages leading to improved PM performance [20,21,22,23,24,25].

Currently, research focused on the relationship between EFT and PM in older adults is limited, with only one study suggesting that EFT, when used as a strategic training method, can enhance PM in this age group [26]. Further investigation into the impact of EFT on PM in older adults is crucial, as it not only deepens our understanding of the mechanisms underlying PM decline with aging but also provides a new avenue for improving PM in this population. According to the Multiprocess Model, the successful execution of PM tasks depends on different cognitive processes, including strategic monitoring and spontaneous retrieval. Strategic monitoring requires individuals to intentionally allocate cognitive resources and actively search for relevant cues while performing PM tasks. This process is resource-intensive, particularly relying on attentional resources and working memory. In contrast, spontaneous retrieval is a process that does not depend on conscious monitoring. It is typically triggered automatically when target cues appear, facilitating the retrieval of intended actions. Spontaneous retrieval is associated with automatic processing and has low cognitive resource demands. The model suggests that age-related differences in EBPM are influenced by the nature of the task: tasks requiring strategic control reveal greater deficits in older adults, whereas tasks relying more on automatic processes exhibit smaller age-related differences [27]. Similarly, the Preparatory Attention and Memory Processes Theory posits that successful completion of PM tasks relies on two core processes: preparatory attention and memory retrieval. The memory process is responsible for activating and retrieving the intended action upon the appearance of a target cue, while the preparatory attention process operates continuously, requiring attentional resources to be allocated in advance for monitoring and detecting target cues. In other words, performing PM tasks consistently demands attentional resources. Given age-related declines in attentional capacity, older adults are more adversely affected when PM tasks require high levels of preparatory attention and memory processing [28]. Previous studies have suggested that the mechanism through which EFT promotes PM lies in the fact that EFT leads to deeper encoding of intentions in memory and establishes an association between the intention and specific visual–spatial contexts [9,10,26]. Based on these theories, it can be hypothesized that EFT may improve PM performance in older adults by reducing the demand for strategic monitoring and enhancing automatic processing. This facilitative effect is expected to be particularly pronounced in older adults.

When examining the improvement of PM in older adults, it is essential to consider the factor of delay interval—the time between intention encoding and task execution. Although PM tasks in real-life settings typically involve longer intervals than those in laboratory experiments, previous research on strategies to enhance PM has rarely addressed the role of delay intervals. Furthermore, the effects of PM improvement strategies might only be immediate, which limits their practical application [28]. Previous studies have shown that older adults’ PM performance is influenced by the length of the delay interval, with better performance at shorter delay intervals [29,30,31,32,33]. Additionally, the Multiprocess Model suggests that longer delay intervals increase the attentional demands and cognitive load of prospective memory tasks, leading to performance decline [29,34,35]. This raises an important question: can EFT help mitigate the decline in PM performance for older adults, especially during longer delay intervals? Earlier research has suggested that EFT can reduce the perceived temporal distance between the present and the future, thereby decreasing impulsive decision-making [36,37,38]. Given EFT’s ability to shorten perceived temporal distance, exploring its potential to improve PM performance over extended delay intervals is both practically and theoretically significant. Therefore, the first aim of this study is to investigate the facilitative effect of EFT on PM in older adults across varying delay intervals.

In most studies, the implementation of EFT involves guiding participants to mentally simulate their upcoming PM tasks, often encouraging them to construct vivid and specific scenarios [10]. Nonetheless, assessing the specificity or vividness of these imagined scenarios can be challenging. Previous research has shown that PM performance is enhanced when PM cues are paired with specific intentions compared to non-specific intentions [3]. For instance, PM tasks with specific cues (e.g., pressing the spacebar when seeing a tiger or a lion) yield better performance than those with non-specific cues (e.g., pressing the spacebar when seeing an animal). Additionally, existing literature indicates a decline in future-thinking-related cognitive abilities among older adults [39,40,41], suggesting that they may struggle to engage their EFT capabilities as effectively as younger adults when left to imagine scenarios independently. This limitation could diminish or negate the potential benefits of EFT for this population. Consequently, the second aim of this study is to investigate how the specificity of EFT influences its facilitative impact on PM in older adults.

In summary, this study consists of two experiments. Experiment 1 examines the effect of EFT on PM in older adults across varying delay intervals, hypothesizing that EFT will facilitate PM in both conditions, with a more pronounced effect in older adults. Experiment 2 investigates whether the specificity of EFT influences its effect on PM in older adults across these delay intervals, hypothesizing that specific EFT will be more effective than non-specific thinking in enhancing PM in older adults and that this facilitative effect will persist even under longer delays. By exploring these relationships, the study aims to identify optimal strategies for enhancing PM performance in this demographic, thereby addressing a critical gap in the existing body of research.

## 2. Experiment 1

### 2.1. Participants

The required sample size was calculated using G*Power 3.1 software with parameters set at f = 0.25, α = 0.05, and 1 − β = 0.8, resulting in a required sample size of 128 participants. A total of 148 participants were recruited, but after excluding those who did not understand the task or had abnormal task responses, we retained 128 participants. Among these, 64 were university students (5 males, 59 females; *M*_age_ = 21.73, *SD*_age_ = 2.46) representing the younger adult population. The older adult group comprised 64 participants (15 males, 49 females; *M*_age_ = 62.37, *SD*_age_ = 5.66), recruited from senior universities and community centers. The Mini-Mental State Examination (MMSE) was used to screen the cognitive level of older adults, with all participants scoring above 24, indicating no cognitive impairment. Additionally, all participants were free from a history of mental illness and did not engage in excessive alcohol consumption or drug use during the testing period. All experiments in this study were approved by the local ethics committee on 11 March 2022, and conducted in line with the Helsinki Declaration. The ethics approval number is 2024016. All participants were tested individually and provided written informed consent.

### 2.2. Design

The study employed a 2 (EFT training: present vs. absent) × 2 (age: younger adults vs. older adults) × 2 (delay interval: 5 min vs. 20 min) between-subjects experimental design. Participants in the EFT training group engaged in approximately 5 min of imagination training during the prospective memory encoding phase, while those in the no-training group received standard verbal instructions. All participants were randomly assigned to their respective experimental condition.

### 2.3. Materials

The experimental program was developed using E-Prime 2.0. Visual stimuli were categorized into ongoing task materials and PM task materials. The ongoing task materials consisted of 600 images drawn from six categories, including fruits, vegetables, clothing, and three additional categories, while the PM task materials comprised 6 images from the animal category.

### 2.4. Control of Ongoing Task Difficulty

Previous research has shown that the difficulty of the ongoing task can influence age differences in PM performance [42]. Older adults might exert more cognitive effort than younger adults when performing the same ongoing task, thereby exacerbating unnecessary age differences. Therefore, this study balanced the ongoing task difficulty across age groups.

A total of 32 older adults (13 males, 19 females; *M*_age_ = 60.50, *SD*_age_ = 6.00) and 16 younger adults (4 males, 12 females; *M*_age_ = 21.00, *SD*_age_ = 2.85) were recruited to assess ongoing task difficulty. Cognitive levels in older adults were screened using the MMSE, with all scoring above 24, indicating no cognitive impairment. All participants had no history of mental illness and did not engage in excessive alcohol consumption or drug use during the testing period.

The ongoing task involved a visual search task in which participants were required to identify an image displayed on the computer and if it matched an initial image category. If a match was present, participants pressed the “F” key; if not, they pressed the “J” key. Younger adults completed the task using a 2×3 grid, while older adults performed both the 2 × 3 and 2 × 2 grid tasks, with accuracy and reaction times recorded. The results indicated no significant difference in accuracy between older adults performing the 2 × 2 grid task (*M* = 0.93, *SD* = 0.03) and younger adults performing the 2 × 3 grid task (*M* = 0.92, *SD* = 0.06), *t* = −1.58, *p* = 0.120, Cohen’s *d* = 0.211. Similarly, reaction times did not differ significantly between older adults on the 2 × 2 grid (*M* = 2432.50, *SD* = 485.90) and younger adults on the 2 × 3 grid (*M* = 2333.00, *SD* = 575.06), *t* = 1.76, *p* = 0.085, Cohen’s *d* = 0.187.

As a result, the formal experiment utilized the 2 × 2 grid visual search task for older adults and the 2 × 3 grid task for younger adults.

### 2.5. Experimental Procedures

Participants were tested individually in a quiet environment. The experimenter first introduced instructions for the ongoing task, requiring participants to perform a visual search task. An initial image was presented at the center of the computer screen, followed by multiple images displayed in a 2 × 3 grid (for younger adults) or a 2 × 2 grid (for older adults). Participants determined whether any of the images matched the category of the initial image. If there was a match, they pressed the “F” key; if not, they pressed the “J” key. After 10 practice trials, participants who responded correctly in 8 or more trials were informed about the PM task. They were instructed to press the spacebar upon encountering PM cues (i.e., animal images) instead of performing categorical judgments. At this point, the EFT training group practiced imagining an unrelated scenario (e.g., checking blood pressure after dinner). After this exercise, participants were instructed to apply the same strategy to the upcoming experiment. They were asked to close their eyes and mentally construct a series of task-related images. For the first 20 s, participants verbally described the imagined scenario, followed by 20 s of silent imagination. This training process took approximately 5 min. The procedure for EFT was based on the design outlined by Kretschmer-Trendowicz et al. [8]. For the non-imagination condition, participants continued viewing task instructions on the computer screen for an equivalent amount of time.

**Figure 1:**
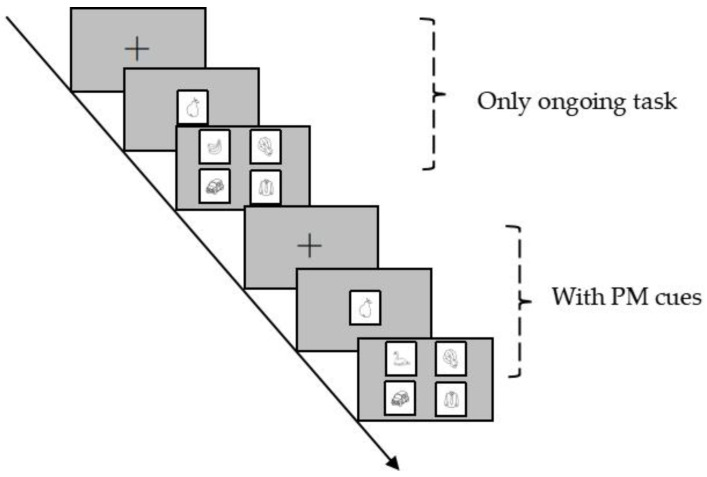
Flowchart of the experiment.

Subsequently, participants entered a delay phase and completed arithmetic tasks involving two-digit addition and subtraction. In the 5 min delay interval condition, participants completed 5 min of arithmetic tasks after receiving PM task instructions, while in the 20 min delay interval condition, they completed 20 min. The formal experiment then commenced, comprising 90 trials, including 10 PM trials and 80 ongoing task trials, with half containing matching category images. At the end of the experiment, participants were asked about the PM task instructions again to exclude those who failed to remember them. The Experimental procedure is shown in Figure 1.

### 2.6. Results Analysis

#### 2.6.1. PM Performance

**Figure 2:**
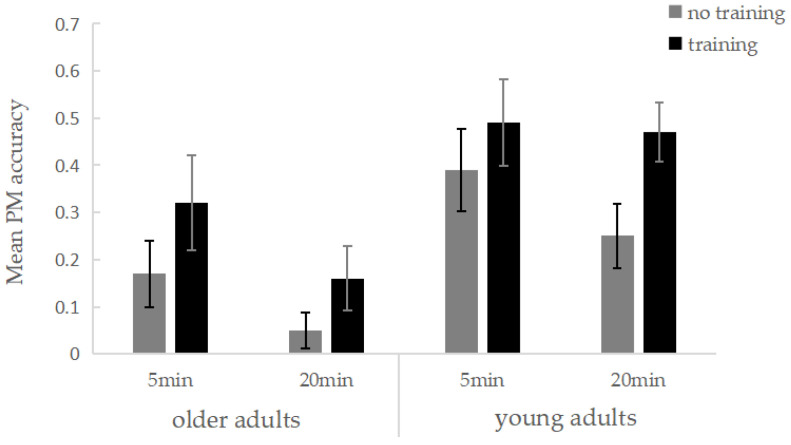
Mean PM accuracy per EFT training, delay interval, and age group condition. Bars represent standard errors.

A 2 (Age: younger adults vs. older adults) × 2 (EFT training: present vs. absent) × 2 (Delay Interval: 5 min vs. 20 min) ANOVA was conducted on PM task accuracy. The results revealed a significant main effect of age, *F*(1, 120) = 18.41, *p* < 0.001, partial *η*^2^ = 0.133, indicating that younger adults outperformed older adults in the PM task. The main effect of delay interval was also significant, *F*(1, 120) = 6.36, *p* = 0.013, partial *η*^2^ = 0.050, with better PM performance observed in the 5 min delay condition compared to the 20 min condition. Furthermore, the main effect of EFT training was significant, *F*(1, 120) = 7.95, *p* = 0.006, partial *η*^2^ = 0.062, showing that the training group performed significantly better on the PM task than the non-training group. No two-way interactions were found between age and delay interval (*p* = 0.921), delay interval and EFT training (*p* = 0.848), or age and EFT training (*p* = 0.692), nor was a three-way interaction observed (*p* = 0.771) (Figure 2).

To further explore the differential impact of EFT training on younger versus older adults, simple effects analyses were conducted. Results indicated that younger adults in the EFT training group demonstrated significantly higher PM accuracy than those in the non-training group (*p* = 0.033). For older adults, the difference between the training and non-training groups approached significance (*p* = 0.078), suggesting that EFT training had a more pronounced facilitative effect on younger adults. In addition, the delay interval had a more pronounced impact on older adults, as their PM performance showed a marginally significant difference between the two delay conditions (*p* = 0.057), with accuracy being lower in the 20 min condition compared to the 5 min condition. In contrast, no significant difference was found between the two delay intervals for younger adults (*p* = 0.106).

A 2 (Age: younger adults vs. older adults) × 2 (EFT training: present vs. absent) × 2 (Delay Interval: 5 min vs. 20 min) ANOVA was conducted on PM task reaction times. A significant main effect of age was found, *F*(1, 120) = 10.36, *p* = 0.002, partial *η*^2^ = 0.079, indicating that younger adults responded more quickly in the PM task than older adults. The main effects of delay interval (*p* = 0.432) and EFT training (*p* = 0.430) were not significant. No two-way interactions were found between age and delay interval (*p* = 0.890), delay interval and EFT training (*p* = 0.916), or age and EFT training (*p* = 0.908), nor was a three-way interaction observed (*p* = 0.557).

The descriptive data of the PM task are shown in Table 1.

#### 2.6.2. Ongoing Task Performance

A 2 (Age: younger adults vs. older adults) × 2 (EFT training: present vs. absent) × 2 (Delay Interval: 5 min vs. 20 min) ANOVA was conducted on the accuracy of ongoing task performance. The results revealed no significant main effects for age (*p* = 0.401), EFT training (*p* = 0.897), or delay interval (*p* = 0.698), suggesting that the difficulty of the ongoing task was effectively equated between the two age groups. Additionally, no two-way interactions were found between age and delay interval (*p* = 0.208), delay interval and EFT training (*p* = 0.974), or age and EFT training (*p* = 0.771), nor was a three-way interaction observed (*p* = 0.948).

A similar 2 × 2 × 2 ANOVA was performed on reaction times for the ongoing task. A significant main effect of age was found, *F*(1, 120) = 7.88, *p* = 0.006, partial *η*^2^ = 0.062, indicating that younger adults responded faster in the ongoing task than older adults. The main effects of delay interval (*p* = 0.687) and EFT training (*p* = 0.545) were not significant. No two-way interactions were found between age and delay interval (*p* = 0.954), delay interval and EFT training (*p* = 0.464), or age and EFT training (*p* = 0.095), nor was a three-way interaction observed (*p* = 0.723).

The descriptive data of the ongoing task are shown in Table 2.

## 3. Experiment 2

### 3.1. Participants

The required sample size was calculated using G*Power 3.1 software with f = 0.25, α = 0.05, and 1-β = 0.8, resulting in a required sample size of 128. A total of 141 participants were recruited, but due to issues such as misunderstanding the task or abnormal task responses, the final valid sample included 128 participants. This consisted of 64 university students (14 males, 50 females; *M*_age_ = 22.08, *SD*_age_ = 2.79) and 64 older adults (13 males, 51 females; *M*_age_ = 61.33, *SD*_age_ = 5.65). Younger participants were recruited from a university, while older participants were recruited from senior universities and communities. The Mini-Mental State Examination (MMSE) was used to screen older adults, all of whom scored above 24, indicating no cognitive impairment. All participants had no history of mental illness and did not excessively consume alcohol or drugs during the testing period.

### 3.2. Design

A 2 (specificity: non-specific vs. specific) × 2 (age: younger vs. older adults) × 2 (delay interval: 5 min vs. 20 min) between-subjects design was employed. The manipulation for the non-specific group was identical to the EFT training group in Experiment 1. In contrast, the specific group underwent imagination training with verbal guidance from the experimenter. After completing the imagination task, both groups rated the vividness of their imagined scenarios on a scale from 1 to 5, with the entire training process taking 5 min. An independent samples t-test showed that the specificity rating for the specific group (*M* = 4.06, *SD* = 0.73) was significantly higher than that for the non-specific group (*M* = 3.02, *SD* = 0.72), *t* = −8.13, *p* < 0.001, Cohen’s *d* = 0.728, indicating the effectiveness of the specificity manipulation. No significant difference in specificity ratings was found between younger and older adults in the specific group (*t* = −1.09, *p* = 0.279, Cohen’s *d* = 1.434).

The materials, procedures, and scoring were identical to those applied in Experiment 1.

### 3.3. Results Analysis

#### 3.3.1. PM Performance

**Figure 3:**
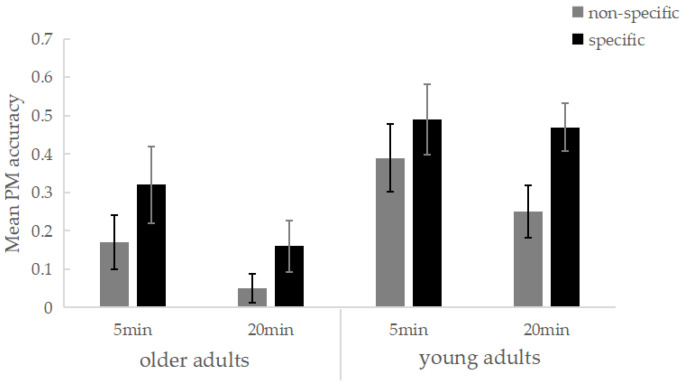
Mean PM accuracy per specificity training, delay interval, and age group condition. Bars represent standard errors.

A 2 (age: younger vs. older adults) × 2 (specificity: non-specific vs. specific) × 2 (delay interval: 5 min vs. 20 min) ANOVA was conducted on PM accuracy. The results revealed a significant main effect of age, *F*(1, 120) = 6.75, *p* = 0.011, partial *η*^2^ = 0.053, indicating that younger adults performed significantly better than older adults. The main effect of delay interval was also significant, *F*(1, 120) = 4.05, *p* = 0.046, partial *η*^2^ = 0.033, showing better PM performance at the 5 min interval compared to the 20 min interval. Furthermore, the main effect of specificity was significant, *F*(1, 120) = 5.58, *p* = 0.020, partial *η*^2^ = 0.044, with the specific group outperforming the non-specific group. No two-way interactions were found between age and delay interval (*p* = 0.520), delay interval and specificity (*p* = 0.251), or age and specificity (*p* = 0.639), nor was a three-way interaction observed (*p* = 0.635). A simple effects analysis revealed that older adults in the specific training group had significantly higher PM accuracy than those in the non-specific group (*p* = 0.025), while no significant differences were observed among younger adults across the two training conditions (*p* = 0.234) (Figure 3). This suggests that specific training was more effective in enhancing PM performance in older adults. Additionally, older adults showed a significant difference between the two delay intervals (*p* = 0.035), with higher PM accuracy at the 5 min interval, whereas no significant difference was found among younger adults (*p* = 0.389).

A 2 (age: younger vs. older adults) × 2 (specificity: non-specific vs. specific) × 2 (delay interval: 5 min vs. 20 min) ANOVA was conducted on PM task reaction times. A significant main effect of age was found, *F*(1, 120) = 19.27, *p* < 0.001, partial *η*^2^ = 0.138, indicating that younger adults had significantly shorter PM reaction times compared to older adults. The main effects of delay interval (*p* = 0.074) and specificity (*p* = 0.181) were not significant. No two-way interactions were found between age and delay interval (*p* = 0.137), delay interval and specificity (*p* = 0.065), or age and specificity (*p* = 0.137), nor was a three-way interaction observed (*p* = 0.598).

The descriptive data of the PM task are shown in Table 3.

#### 3.3.2. Ongoing Task Performance

A 2 (Age: younger adults vs. older adults) × 2 (Specificity: non-specific vs. specific) × 2 (Delay interval: 5 min vs. 20 min) ANOVA was conducted on ongoing task accuracy. The results revealed no significant main effects for age (*p* = 0.382), specificity (*p* = 0.842), or delay interval (*p* = 0.720). Additionally, no two-way interactions were found between age and delay interval (*p* = 0.842), delay interval and specificity (*p* = 0.873), or age and specificity (*p* = 0.450), nor was a three-way interaction detected (*p* = 0.633).

Similarly, a 2 (Age: younger adults vs. older adults) × 2 (Specificity: non-specific vs. specific) × 2 (Delay interval: 5 min vs. 20 min) ANOVA was performed on ongoing task reaction times. The analysis showed no significant main effects for age (*p* = 0.096), specificity, or delay interval (*p* = 0.416). Additionally, no two-way interactions were found between age and delay interval (*p* = 0.524), delay interval and specificity (*p* = 0.513), or age and specificity (*p* = 0.836), nor was a three-way interaction detected (*p* = 0.409).

The descriptive data of the ongoing task are shown in Table 4.

## 4. General Discussion

This study investigates the potential of EFT to enhance PM performance in older adults, while accounting for differences in ongoing task difficulty between older and younger participants. By drawing on the Multiprocess Model, the Preparatory Attention and Memory Processes Theory, and the unique characteristics of EFT in supporting delayed intentions, this research is the first to examine the facilitative effect of specific EFT training on PM in older adults. Additionally, it explores how EFT improves PM across different delay intervals, addressing a notable gap in the literature. Given the age-related declines in cognitive abilities, particularly in tasks requiring strategic control and sustained attention, understanding how EFT aids PM is crucial for developing effective interventions that support the everyday cognitive functioning of older adults.

Firstly, this study investigates age-related differences in PM. Both Experiment 1 and Experiment 2 confirmed age-related declines in PM performance among older adults compared to younger adults, aligning with findings from previous research [5,6]. This decline can be attributed to the fact that completing PM tasks requires attentional resources, which tend to diminish with age. As a result, older adults often perform worse on PM tasks, especially those that demand strategic monitoring and focused attention, due to reductions in attention and executive control functions [43]. These findings support the principles of the Multiprocess Model and the Preparatory Attention and Memory Processes Theory.

Secondly, the research focuses on the improvement of older adults’ PM through EFT as an encoding strategy. Experiment 1 found that EFT training had a significant promoting effect on PM, consistent with previous studies [9,10,26]. EFT deepens the encoding of intentions and strengthens the connection between cues and actions, thereby enhancing PM performance. Furthermore, in Experiment 1, the PM task accuracy of the EFT training group was significantly higher than that of the non-training group, while there was no significant difference in the ongoing task accuracy between the two groups. This suggests that the promoting effect of EFT on PM does not require additional cognitive resource support, providing evidence for its role in automated processing. However, for older adults, the training group showed less improvement compared to younger adults, possibly due to age-related declines in future scenario thinking ability [39,40,41]. For example, Addis et al. [39] proposed that the aging of future thinking abilities may prevent older adults from obtaining the same level of benefit from EFT as younger adults. These findings highlight the necessity of further research on how the specificity of EFT affects its effectiveness in improving PM, especially in older adults.

The study then explored the promoting effect of EFT training on older adults’ PM. Experiment 2 confirmed that specific EFT training had a greater promoting effect on PM, aligning with earlier research on the role of specific encoding in enhancing prospective memory [44,45,46]. It can also be speculated that specific EFT training forms stronger links between intentions and specific visual-spatial contexts, leading to deeper encoding of intentions in memory. Unlike Experiment 1, in Experiment 2, we found that specific EFT training provided greater benefits for older adults’ PM. We hypothesize that, due to the overall decline in cognitive abilities in older adults, simple EFT training has limited effects on PM improvement. More targeted and specific EFT methods are needed to achieve better outcomes; specific EFT training may help compensate for the decline in future thinking abilities in older adults. Additionally, Experiment 2 did not find a main effect of ongoing task specificity, indicating that older adults benefited from specific EFT training without consuming additional cognitive resources. This aligns with the findings of Scullin et al. [47], who discovered through a thinking probe program that generating specific prospective cue examples improved performance without increasing the burden of ongoing tasks, providing evidence for automatic retrieval support. It is evident that specific EFT training still improves older adults’ PM by enhancing automated processes.

A noteworthy finding was that in Experiment 1, after a 20 min delay interval, the EFT training group performed significantly better than the non-training group (*p* = 0.040), whereas this effect was not observed at the 5 min interval (*p* = 0.082). This result indicates that EFT training is especially effective in enhancing PM performance over longer delay intervals. This might be related to EFT reducing the perceived temporal distance between present and future [36,37,38], making future intentions feel more immediate and thereby improving performance on prospective memory tasks. Nonetheless, in Experiment 2, under the 20 min delay, there was no significant difference between specific and non-specific training (*p* = 0.395). According to the Temporal Construal Theory [48], as the temporal distance to a future event increases, the event is more likely to be represented in an abstract manner, emphasizing its general perceptual features rather than its specific, incidental details. This concept can be understood through everyday experience—when anticipating an activity that is scheduled for a distant future time, we tend to envisage only the key aspects of the task rather than meticulously planning out every detail. Spreng and Levine [49] argued that if EFT is intended to coordinate behavior, then creating detailed representations of unrelated events over a long future period can be cognitively inefficient. This suggests that EFT training facilitates PM retrieval over longer delay intervals, even without requiring specific operationalization. In the present study, the long delay interval was set at 20 min. Given that PM tasks in everyday life typically involve much longer intervals, future research—particularly studies conducted in naturalistic settings—should explore the effects of EFT over extended time intervals, such as several hours or even an entire day.

Furthermore, the oral description of the imagined scenario in the first 20 s might have inadvertently encouraged participants to use verbal representations, potentially influencing the integrity of the EFT effect. It is important to note that using implementation intentions to enhance PM also involves verbal representations. This strategy usually requires participants to rehearse statements like, “If situation X occurs, then I will execute intention Y” [50]. In this study, participants’ verbalization during the first 20 s of imagination was somewhat analogous to this rehearsal of implementation intentions. Therefore, future research is advised to methodologically distinguish between the linguistic representation and the imaginative representation of EFT. This differentiation can also be achieved by manipulating the PM task to discern the cognitive processing modes activated by EFT. For instance, future studies could investigate whether the effects of EFT differ between perceptual and semantic PM tasks, thereby providing a more in-depth understanding of the facilitative mechanisms underlying EFT.

## 5. Conclusions

This study examined the effectiveness of EFT in enhancing older adults’ PM, focusing on its impact across different delay intervals and encoding strategies. Consistent with prior research, the study confirmed the age-related decline in PM, highlighting its connection to reductions in cognitive resources. While older adults experience challenges with PM, EFT training—particularly specific EFT training—proved beneficial in mitigating this decline. Notably, EFT demonstrated a significant positive effect on PM tasks involving longer delay intervals. However, as the delay interval increased, the effectiveness of specific EFT training diminished. These results underscore the relevance of the Multiprocess Model and the Preparatory Attention and Memory Processes Theory in explaining EFT’s benefits. Furthermore, the findings support the use of EFT training as a practical tool to enhance PM performance in older adults, offering potential applications for future interventions.

## Figures and Tables

**Table 1:** The PM performance of older and younger adults under different delay intervals and different EFT training conditions (M and SD).

Age Group	Accuracy				Reaction time			
	Training		No Training		Training		No Training	
	5 min	20 min	5 min	20 min	5 min	20 min	5 min	20 min
Younger adults	0.49 (0.37)	0.47 (0.25)	0.39 (0.35)	0.25 (0.27)	2415.17 (714.14)	2398.12 (571.33)	2352.93 (612.79)	2237.26 (462.20)
Older adults	0.32 (0.40)	0.16 (0.27)	0.17 (0.28)	0.05 (0.15)	2806.17 (572.69)	2640.53 (567.32)	2647.27 (579.30)	2623.61 (504.92)

Note: training: episodic future thinking; no training: the absence of episodic future thinking.

**Table 2:** The ongoing task performance of older and younger adults under different delay intervals and different EFT training conditions (M and SD).

Age Group	Accuracy				Reaction Time			
	Training		No Training		Training		No Training	
	5 min	20 min	5 min	20 min	5 min	20 min	5 min	20 min
Younger adults	0.92 (0.05)	0.92 (0.02)	0.92 (0.03)	0.91 (0.05)	2521.02 (625.11)	2702.84 (612.80)	2429.21 (617.19)	2263.52 (455.17)
Older adults	0.92 (0.07)	0.94 (0.04)	0.92 (0.07)	0.93 (0.06)	2709.14 (616.44)	2712.61 (612.69)	2864.43 (742.43)	2786.82 (551.74)

Note: training: episodic future thinking; no training: the absence of episodic future thinking.

**Table 3:** The PM performance of older and younger adults across different delay intervals and specificity conditions (M and SD).

Age Group	Accuracy				Reaction Time			
	Specific		Non-Specific		Specific		Non-Specific	
	5 min	20 min	5 min	20 min	5 min	20 min	5 min	20 min
Younger adults	0.56 (0.37)	0.44 (0.39)	0.41 (0.41)	0.36 (0.36)	2064.80 (539.28)	2301.27 (742.15)	2379.37 (660.24)	2074.09 (711.72)
Older adults	0.50 (0.33)	0.24 (0.29)	0.23 (0.30)	0.17 (0.27)	2986.39 (554.20)	2764.94 (612.05)	2789.43 (788.84)	2265.41 (434.01)

Note: training: episodic future thinking; no training: the absence of episodic future thinking.

**Table 4:** The ongoing task performance of older and younger adults across different delay intervals and specificity conditions (M and SD).

Age Group	Accuracy				Reaction Time			
	Specific		Non-Specific		Specific		Non-Specific	
	5 min	20 min	5 min	20 min	5 min	20 min	5 min	20 min
Younger adults	0.92 (0.06)	0.92 (0.04)	0.91 (0.03)	0.92 (0.05)	2333.00 (575.06)	2481.71 (852.89)	2565.49 (650.03)	2376.60 (804.19)
Older adults	0.92 (0.03)	0.93 (0.05)	0.93 (0.04)	0.93 (0.05)	2667.38 (539.39)	2482.22 (332.99)	2758.46 (723.46)	2612.65 (505.04)

Note: specific: specific episodic future thinking; non-specific: non-specific episodic future thinking.

## Data Availability

The data can be accessed on 18 October 2024 at https://osf.io/wpbn5/.
